# Influences of Water Glass and Sodium Methyl Silicate Combined Treatment on Recycled Coarse Aggregate and Concrete Made with It

**DOI:** 10.3390/ma18225223

**Published:** 2025-11-18

**Authors:** Jinming Yin, Aihong Kang, Changjiang Kou

**Affiliations:** 1 Taizhou Institute of Science and Technology, Nanjing University of Science and Technology, Taizhou 225300, China; 2 College of Civil Engineering and Transportation, Yangzhou University, Yangzhou 225100, China

**Keywords:** recycled coarse aggregate, recycled aggregate concrete, water glass, sodium methyl silicate

## Abstract

The increasing generation of construction and demolition waste (CDW) and the overexploitation of natural aggregates (NA) have necessitated sustainable solutions for recycled aggregate concrete (RAC). This study proposes an innovative inorganic–organic combined modification method using water glass (WG) and sodium methyl silicate (SMS) to enhance the performance of recycled coarse aggregate (RCA) and RAC. A comprehensive experimental program was conducted, including crushing value tests, capillary water absorption, compressive and splitting tensile strength analysis, nanoindentation and Fourier transform infrared spectroscopy (FTIR). The results demonstrated that the combined treatment of 40% WG and 10% SMS significantly improved the RCA properties, reducing water absorption by up to 46.47% and increasing the compressive strength of the RAC by 34.8%. Through mechanistic analysis, it was found that after treatment with SMS solution, a hydrophobic film formed on the surface of the RCA, thereby preventing the transmission of moisture. The interface transition zone between the RCA and the new cement mortar was enhanced, consequently improving the mechanical properties of the RAC. This study contributes to improving the properties of recycled aggregate and recycled aggregate concrete, and to the understanding of the mechanism of combined modification.

## 1. Introduction

The rapid urbanization and urban renewal processes have generated a large amount of construction and demolition waste (CDW), especially in developing countries [1,2,3]. In China, the annual production of construction waste exceeded 3 billion tons [4]. To manage these CDW, a significant amount of land is required, which also leads to adverse environmental impacts [5]. At the same time, the construction industry consumes a large amount of natural aggregates (NA). Globally, about 50 billion tons of NA are consumed annually, leading to significant impacts on the ecological environment [6,7]. Hence, utilizing CDW to produce recycled aggregate (RA) as a substitute for NA not only addresses the issues caused by CDW but also mitigates the problems associated with the extraction of NA [8,9]. However, due to the adhered mortar, RA exhibits higher water absorption, lower strength, and lower density. As a result, RAC performs worse than NAC [10,11,12].

To enhance the performance of RA and RAC, numerous studies have been conducted. Removing the old mortar of RA is an effective approach. By placing RA in a specialized machine, RA can be forced to move quickly, and the movement of RA causes impact and friction between the RA and the machine, as well as between the RAs themselves. This process helps to remove or partially remove the adhered old mortar [13,14]. According to [15,16], heating RA can improve the effectiveness of removing the old mortar. Soaking RA in various acid solutions, including acetic acid, tannic acid, hydrochloric acid and sulfuric acid, is another method to remove the old mortar [17,18,19]. Acid solutions, while dissolving the old mortar, can also cause damage to the NA. The removal of adhered mortar through mechanical or thermal methods results in increased energy consumption and carbon emissions, whereas the use of acid soaking for mortar removal poses risks of environmental pollution [20,21]. Therefore, the development of efficient strengthening methods with minimal environmental impact is of great importance.

Surface treatment represents another approach to strengthening RA. Since this method does not require the removal of adhered mortar, it results in a lower environmental impact and has consequently attracted significant attention from researchers. A commonly used surface treatment method for enhancing the performance of RA involves encapsulating them with high-strength or dense materials. Typical encapsulating materials include cement-based compounds and polymers, such as polyvinyl alcohol (PVA). The cement-based material, prepared as a slurry, is applied to the RA surface by immersion or spraying. After hardening, a dense coating layer with relatively high strength is formed, which improves the surface smoothness and, consequently, the overall performance of the RA. However, the hardened cementitious layer is not effective in preventing moisture ingress; as a result, this method does not significantly reduce, and may even increase, the water absorption of RA [21,22,23]. Polymer materials are typically applied to modify recycled aggregates (RA) in solution form. The polymer molecules form a dense film on the RA surface, effectively improving surface roughness and reducing water absorption. However, if the thickness of the film is not properly controlled, the polymer layer may interfere with the bonding between the RA and the new cement matrix, which could negatively affect the performance of the RAC [24,25,26,27]. Another surface treatment technique involves the penetration of active substances into the surface layer of the adhered old mortar on recycled aggregates (RA). The active substances react with the mortar, and the resulting products fill the internal pores, thereby enhancing the strength of the RA and reducing its water absorption. Typical strengthening agents include nanomaterials, water glass and CO_2_ [7,20,28,29,30,31,32,33]. However, nanomaterials are costly, and CO_2_ treatment requires specialized equipment, limiting their practical application. In contrast, water glass treatment—especially by immersion—has been widely adopted, due to its simplicity and applicability. Nonetheless, although water glass treatment can partially mitigate the high water absorption of RA, capillary pores within the old mortar remain channels for moisture ingress.

Owing to the energy consumption, carbon emissions, and environmental pollution associated with the removal of old mortar, methods for strengthening old mortar have received more attention. Nevertheless, further research is warranted to develop optimized treatment strategies that synergistically improve the performance of both RA and RAC, particularly through combined treatment methods utilizing multiple reinforcing agents. Furthermore, the mechanism by which combined treatment methods affect RCA and RAC requires further investigation. In this paper, an inorganic–organic combined modification method, using water glass (WG) and sodium methyl silicate (SMS), was proposed, aiming to enhance the strength and durability of RAC. The influences and mechanisms of the combined method on RA and RAC were verified. This work provides an effective approach for enhancing the performance of RA and RAC and offers a better understanding of the mechanisms through which the treatment method influences both the RA and the RAC.

## 2. Materials and Methodology

### 2.1. Materials

#### 2.1.1. RCA

**Figure 1:**
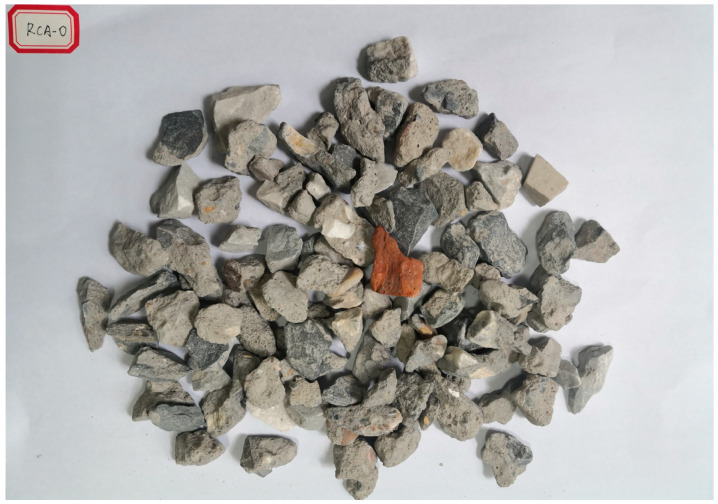
Raw RCA.

In this study, recycled coarse aggregate (RCA), supplied by Yangzhou Huimin Renewable Resources Co., Ltd. (Yangzhou, China), was mainly made of concrete, as shown in Figure 1. Because of the adhered old mortar, the surface of the RCA was rough, porous, and had cracks. According to the Chinese code of *Recycled coarse aggregate for concrete* (GB/T 25177-2010) [34], the main technical specifications of the RCA are listed in Table 1. Evidently, the performance of the RCA was mainly limited by its water absorption. To improve the RCA, reducing the water absorption is more effective.

#### 2.1.2. Water Glass

A liquid water glass (WG), provided by Nanchang Hongshun Industrial Co., Ltd. (Nanchang, China), was used in this study. The contents of Na_2_O and SiO_2_ were 7.9% and 24.2%, respectively. The module and Baume degree were 3.2 and 37, respectively. To obtain WG solutions with different concentrations, tap water was added to the liquid WG and the concentrations of the solutions were controlled based on mass percentage.

#### 2.1.3. Sodium Methyl Silicate

**Figure 2:**
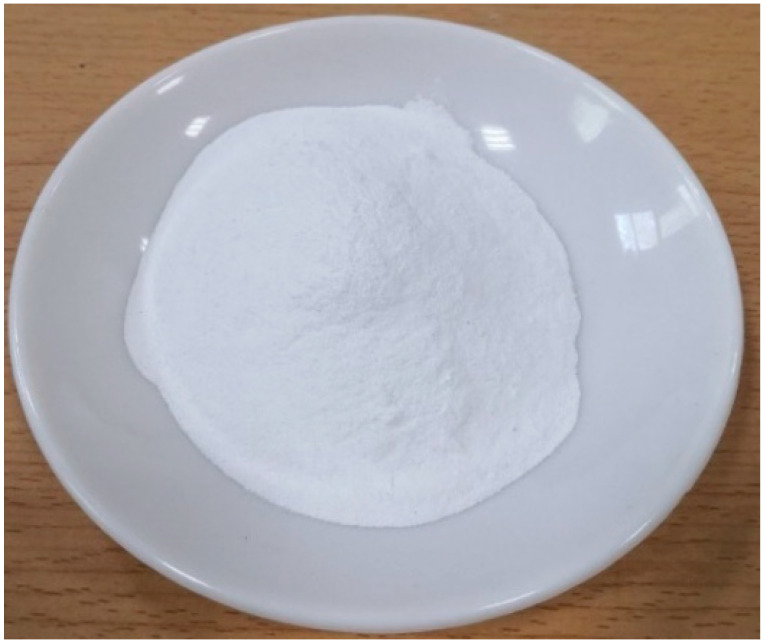
Image of sodium methyl silicate [36].

A powdered sodium methyl silicate (SMS) (as shown in Figure 2), purchased from Jinan Xingchi Chemical Co., Ltd. (Jinan, China), was used to make SMS solutions with varying concentrations. The solid content and alkali content were 98% and 29%, respectively.

#### 2.1.4. Cement

An ordinary Portland cement with a strength grade of 42.5 was used, which was produced by Taizhou Yangwan Conch Cement Co., Ltd. (Taizhou, China). The chemical composition of the cement is listed in Table 2.

#### 2.1.5. Natural Aggregate

River sand was used as a natural fine aggregate (NFA). The fineness modulus of the sand was 2.6. The particle size of the natural coarse aggregate (NCA) was no more than 19 mm. The apparent density, water absorption and crushing value of the NCA were 2745.1 kg/m^3^, 0.46% and 8.15%, respectively.

### 2.2. Test Methods

#### 2.2.1. RCA Treatment and Test

(1) RCA Treatment method

**Figure 3:**
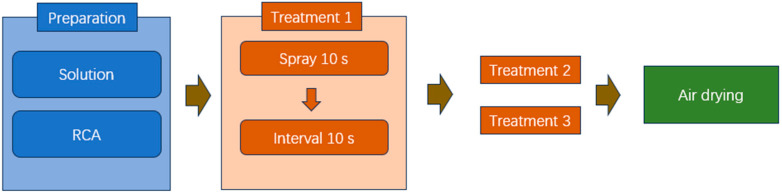
Single-solution treatment process.

**Figure 4:**
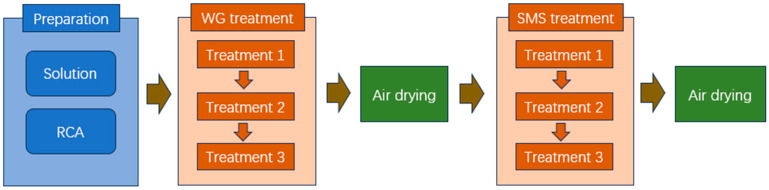
Two-solution treatment process.

To save materials, the spraying method was adopted to treat RCA, and the schematic of the spraying system is shown in [35]. A single spraying cycle consists of spraying solution for 10 s, followed by a 10 s interval. To ensure thorough absorption of the RCA, three spraying cycles were performed. During the spraying treatment, the RCA was kept in a vibrating state. The treatment process, using either WG solution or SMS solution alone, is shown in Figure 3. The treatment process using both WG and SMS solutions for RCA is shown in Figure 4. The test scheme is listed in Table 3. The preparation of all solutions was controlled according to the mass percentage.

(2) Performance tests on RCA

Based on the Chinese code of *Recycled coarse aggregate for concrete* (GB/T 25177-2010) [34], the crushing value test and water absorption test were conducted to evaluate the influences of the treatment method on the RCA.

(3) Micromorphology test on RCA

The scanning electron microscope (SEM) was used to analyze the impact of different treatment methods on the morphology of RCA. GeminiSEM 300 (Carl Zeiss, Oberkochen, Germany) was used, and the acceleration voltage was 10 kV. The image was captured at a magnification of 2000×.

#### 2.2.2. Concrete Preparation and Test

##### Preparation of Concrete

The designed strength grade of the concrete was C40, with a target slump class of S3 (100–150 mm). The proportion of concrete is presented in Table 4. For natural aggregate concrete (NAC), air-dried natural aggregate (NA) was used. For RAC, NA was completely replaced by treated or untreated RCA. Owing to the high water absorption of RCA, their direct incorporation into the concrete mix often leads to reduced workability. To ensure the workability of the RAC mix, both the untreated RCA and WG solution-treated RCA were soaked in water for 24 h and used in a saturated dry-surface state. For the combined treatment method of the WG solution and SMS solution, as well as the single treatment method using the SMS solution, the treated RCA was used to prepare the RAC immediately after spraying the SMS solution. The test scheme is listed in Table 5.

##### Performance Tests of Concrete

(1) Compressive strength and splitting tensile strength

Based on the Chinese code of *Standard for test methods of concrete physical and mechanical properties* (GB/T 50081-2019) [37], the compressive strength and splitting tensile strength of concrete were tested at 28 days. For each group, at least three specimens were prepared, each in the form of a cube with a side length of 100 mm.

(2) Capillary water absorption

According to ASTM C1585-13 [38,39], the capillary water absorption test was conducted. The capillary water absorption per unit area was calculated with the following equation:

(1) $$I=\frac{m_{\mathrm{wt}}}{a\cdot d}$$

where *I* is capillary water absorption per unit area, mm; $m_{\mathrm{wt}}$ is the mass of water absorbed by the concrete at time *t*, g; *a* is the area through which concrete sample absorbs water, mm^2^; and *d* is the density of water, g/mm^3^.

Linear fitting was used to determine the relationship between *I* and $\sqrt{t}$, as shown in Equation (2). The slopes of the initial 6 h and remaining time represent the initial water absorption rate (*S*_i_) and the second-stage water absorption rate (*S*_s_), respectively.

(2) $$I=S\sqrt{t}+b_{1}$$

where *S* is the water absorption rate, mm/s^0.5^ and *b*_1_ is a fitting parameter, mm.

(3) Coulomb electric flux

The coulomb electric flux test can reflect the ability of concrete to resist chloride ion penetration under saturated conditions. In this study, based on the Chinese code of *Standard for test methods of long-term performance and durability of ordinary concrete* (GB/T 50082-2009) [40], the coulomb electric flux test was conducted at 28 days. Using the following equations, the coulomb electric flux was calculated.

(3) $$Q_{x}=900(I_{0}+2I_{30}+2I_{60}+\ldots +2I_{t}+\ldots +2I_{330}+{\;I}_{360})$$

(4) $$Q_{s}={\;Q}_{x}\;\times \;(95/100{)}^{2}$$

where $Q_{x}$ is the coulomb electric flux of the sample with a 100 mm diameter, C; $I_{0}$ is the initial current, A; $I_{t}$ is the current at time *t* (the interval time is 30 min), A; and $Q_{s}$ is the coulomb electric flux of the sample with a 95 mm diameter, C.

(4) Contact Angle Test

The contact angle test was conducted to characterize the influence of different treatment methods on the wettability of old mortar. First, old mortars were treated using the 40% WG solution (O_W), 10% SMS solution (O_M) and the combined method (O_WM), respectively. Next, the contact angle was measured, using an instrument manufactured by Shanghai Zhongchen Digital Technology Equipment Co., Ltd. (Shanghai, China).

(5) Nanoindentation

**Figure 5:**
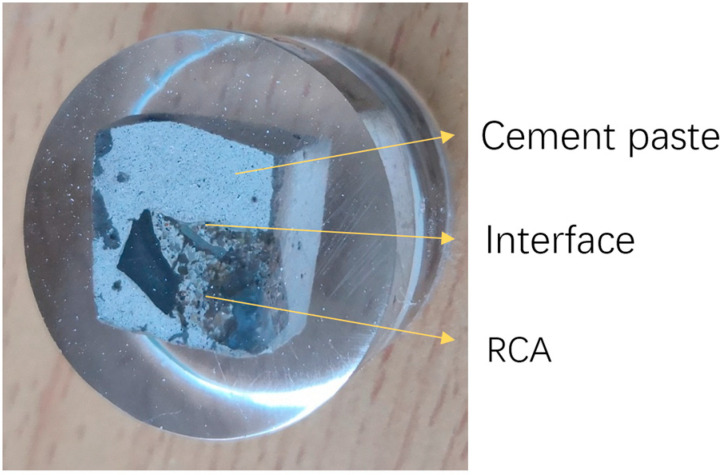
A typical sample for NI tests [36].

**Figure 6:**
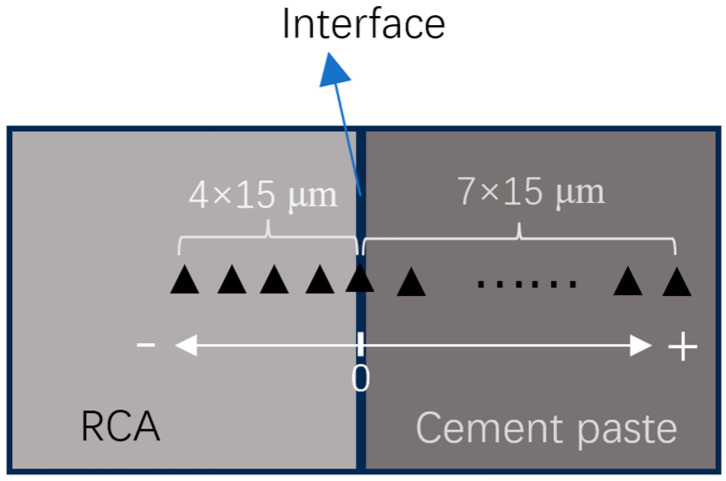
Arrangement of NI points [36].

Nanoindentation (NI) was employed to evaluate the effect of different RCAs on the interfacial transition zone between RCA and new mortar. First, the treated RCAs were selected before the preparation of RACs. Next, the selected RCAs were placed in 3 cm cube molds, and a 0.5 water-to-cement ratio (*w*/*c*) paste was poured into the molds. After 1 day, the samples were demolded and cured for 28 days. Then, the samples were cut and embedded in resin. Finally, the samples were ground and polished. A typical sample is shown in Figure 5. The arrangement of NI points is depicted in Figure 6. The scheme of the NI test is listed in Table 6.

(6) Fourier Transform Infrared Spectrum

Fourier transform infrared spectrum (FTIR) was employed to characterize the influences of different treatment methods on old mortars and new paste. The test scheme is listed in Table 7. For the new paste, 10% SMS solution was used to partially replace the water when the new mortars were prepared. Then, the old mortars and cured new mortar were ground into powder. The instrument manufactured by PerkinElmer (Springfield, IL, USA) was adopted.

## 3. Results and Discussion

### 3.1. Morphology Analysis of RCA

**Figure 7:**
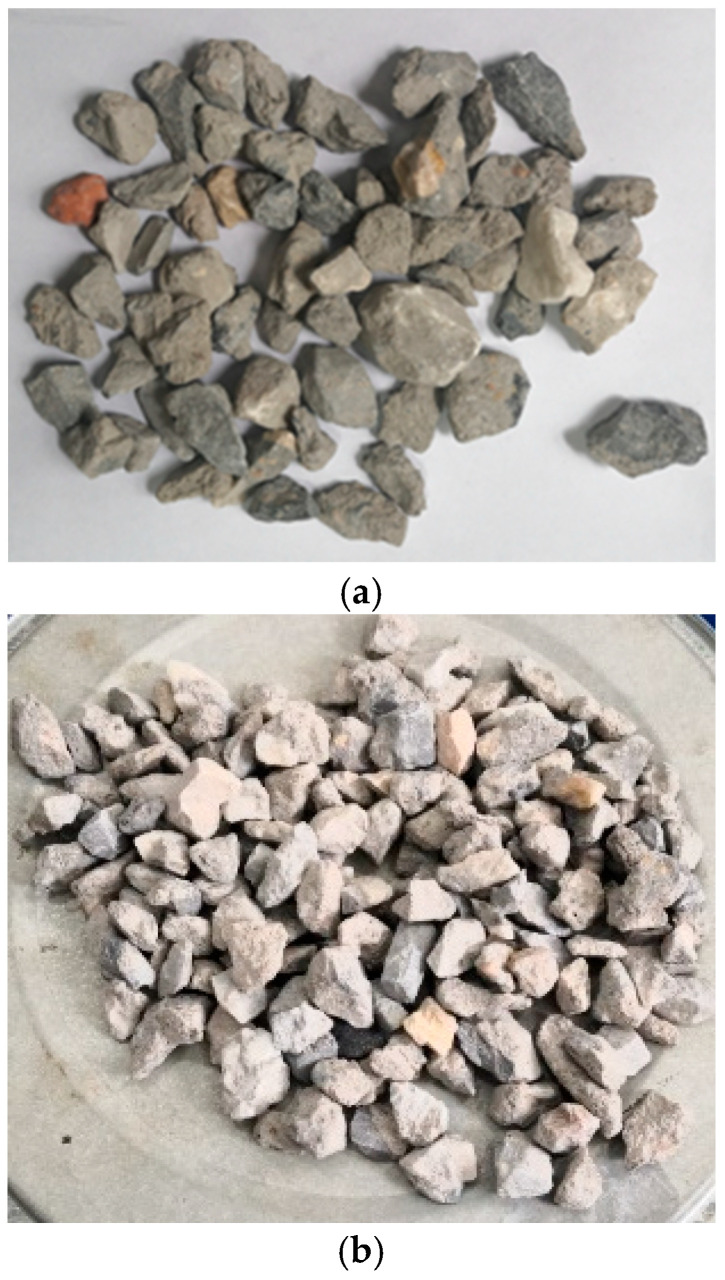
Macro morphology of the RCA. (**a**) WG treated RCA. (**b**) WG and SMS treated RCA.

**Figure 8:**
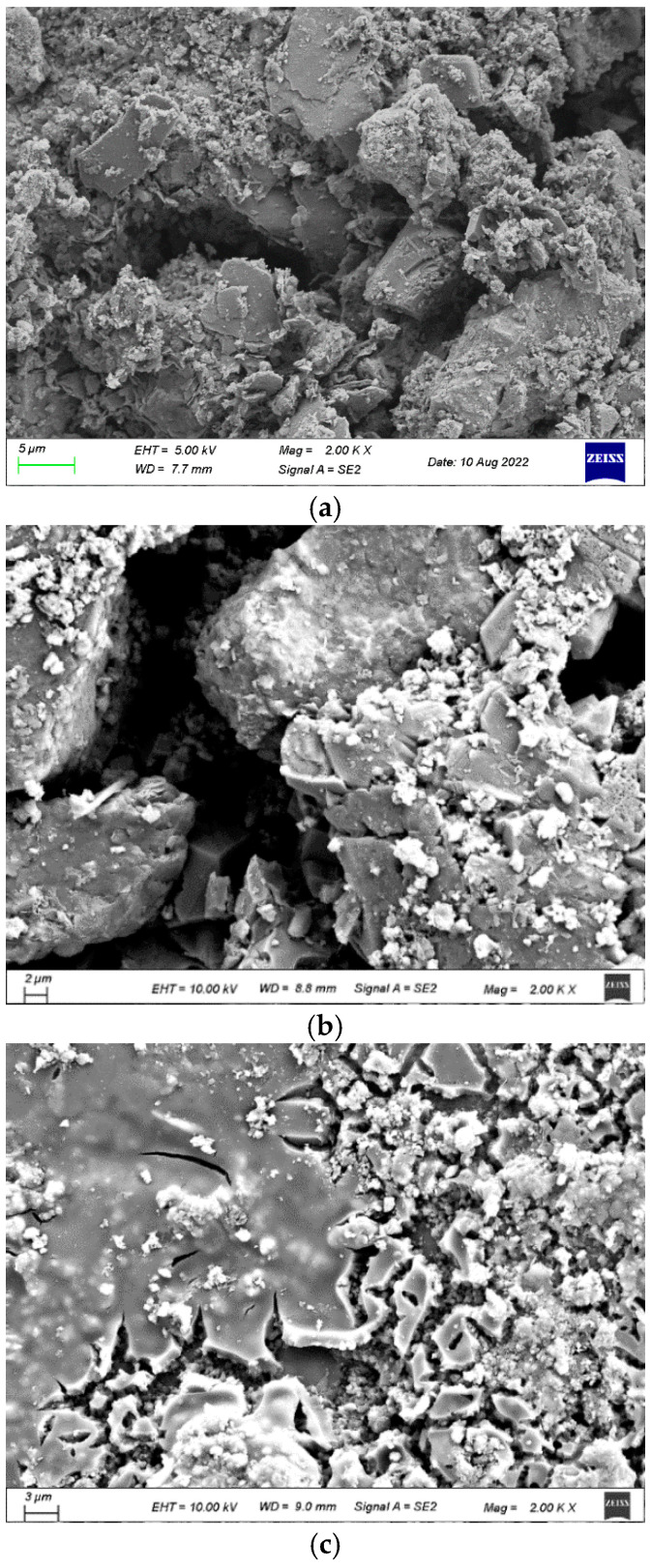
SEM images of untreated and treated RCA. (**a**) SEM image raw RCA. (**b**) SEM image of RCA treated with SMS. (**c**) SEM image of RCA treated with WG and SMS.

The treated RCAs are shown in Figure 7 and Figure 8. In terms of macro morphology, the surfaces of the RCAs did not form a distinct coating layer, and the treated RCAs remained rough and porous. It meant that during the spraying process, the WG solution and SMS solution mainly infiltrated the RCA.

In terms of micro morphology, pores and cracks could be clearly observed in the SMS-solution-treated RCA, showing no significant difference from the raw RCA. As reported in [41], a transparent molecular film formed through the reaction between SMS and RCA, which could not be observed. For the WG-solution-treated RCA, a gel layer could be observed, which was consistent with [42]. Nevertheless, the gel layer did not completely cover the recycled aggregate, which still permitted the ingress of water into the RCA.

### 3.2. Crushing Value and Water Absorption of RCA

**Figure 9:**
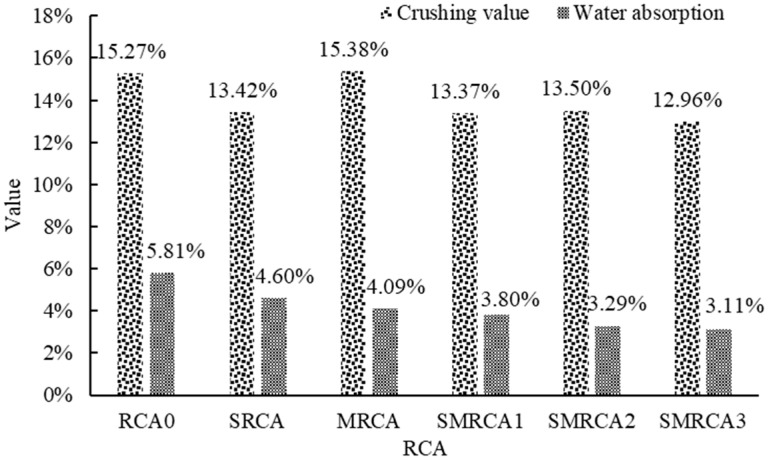
Test results of crushing value and water absorption.

The crushing value results and water absorption results are shown in Figure 9. Apparently, the strength of the RCA was improved by the WG solution, and the crushing value decreased by 12.11% when the 40% WG solution was used. During the spraying process, the WG solution penetrated the RCA, and then C-S-H was generated, due to the reaction between the WG and the old mortar. The pores and cracks of the old mortar could be filled, thereby improving the RCA’s strength. Similar results were also reported in [43]. For the SMS-solution-treated RCA, the crushing value was not significantly affected. This is because only a molecular film was formed on the treated RCA’s surface, which did not affect its strength.

Compared with the untreated RCA, the water absorption of the WG-solution-treated RCA decreased by 20.83%. As reported in [1], this phenomenon is attributed to the formation of C-S-H through the reaction between WG and the old mortar, as well as the formation of the gel layer on the surface. Furthermore, the interaction between the C-S-H gel, formed by the reaction of the water glass with the old mortar, and water molecules restricts the free movement of water within the pores, which further reduces the water absorption of the treated RCA [44]. The water absorption of the MRCA decreased by 29.60%. This is because SMS is a waterproof material which could form a hydrophobic layer on the cement-based material [45]. When WG and SMS were used in combination, the water absorption was further reduced. Specifically, for SMRCA1, SMRCA2 and SMRCA3, the water absorption decreased by 34.60%, 43.37% and 46.47%, respectively. This synergistic effect demonstrates that WG and SMS collaboratively enhance the water-blocking performance, effectively inhibiting moisture infiltration. Mechanistically, WG reacts to form calcium silicate hydrate (C-S-H) and gel, filling pores and cracks within the RCA. Simultaneously, SMS creates a hydrophobic layer on the RCA surface. As the concentration of the SMS solution increased, the water absorption of the treated RCA decreased. As the SMS solution concentration increased, the water absorption of the treated RCA progressively decreased. At a 10% SMS concentration, the hydrophobic layer achieved optimal coverage, resulting in a significant reduction in water absorption. Beyond this concentration, the improvement in water absorption became marginal, indicating the saturation of the hydrophobic effect.

### 3.3. Contact Angle

**Figure 10:**
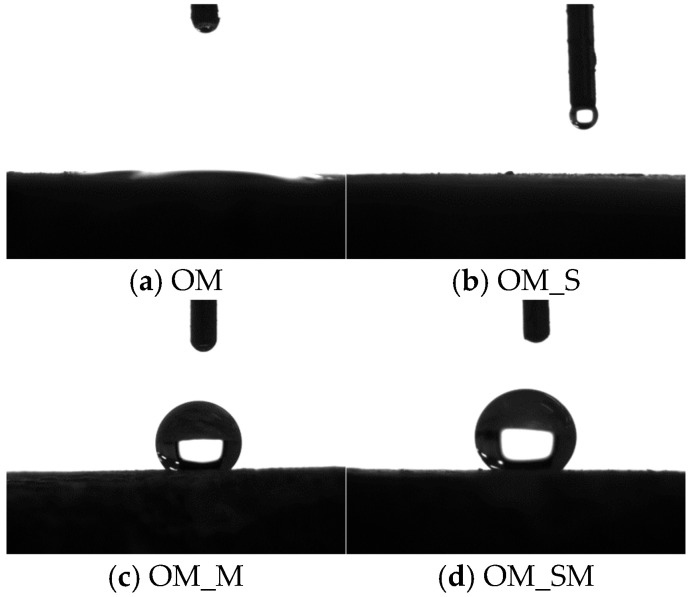
Test results of contact angle.

The contact angle results are presented in Figure 10. The results show that both the untreated old mortar and WG-solution-treated old mortar were hydrophilic, while the SMS-solution-treated old mortar and combined-treated old mortar were hydrophobic. This is because the reaction between SMS and the old mortar lead to the formation of a hydrophobic film on the surface of the old mortar. The contact angles of OM_M and OM_SM were greater than 120°. According to the Young–Laplace equation (Equation (5)) [46], the capillary suction was negative, indicating that water could not penetrate the old mortar, which consequently reduced the water absorption of the treated RCA. For the WG-solution-treated RCA, the reduction in water absorption was due to the sealing effect of the gel layer, as shown in Figure 9.

(5) $$P_{gl}=\frac{2\gamma \cos\theta }{r}$$

### 3.4. Compressive Strength and Splitting Tensile Strength of Concrete

**Figure 11:**
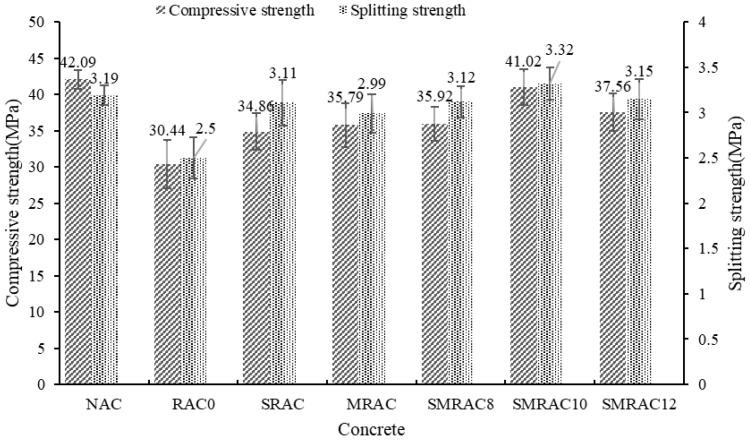
Test results of compressive strength and splitting tensile strength.

The compressive strength and splitting tensile strength results are shown in Figure 11. When the NCA was replaced by the untreated RCA, the compressive strength and splitting tensile strength decreased by 27.7% and 21.6%, respectively. Similar results were also reported in [47]. This means that the use of RCA negatively affected the strength of the RAC. According to the failure mode, both interface failure and RCA failure were observed, particularly at the interface between the old mortar and the new mortar. Therefore, improving the strength of the RCA and the interface are beneficial for enhancing the strength of the RAC.

When the RCA was treated with the WG solution, the compressive strength and splitting tensile strength of the SRAC increased by 14.5% and 24.4%, respectively. A similar result was also reported in [48]. On one hand, the strength of the RCA was enhanced through the WG solution treatment. On the other hand, in the RAC, the water content was reduced due to the lower water absorption of the treated RCA. Therefore, the strength of the SRAC was higher than the RAC0. According to Alqarni A S [49], the enhancement induced by the WG solution treatment can be attributed to the reaction between the WG and the old mortar, which forms a dense layer on the surface of the RCA.

For MRAC, the SMS-solution-treated RCA was used to replace the NCA, and the compressive strength and splitting tensile strength increased by 17.6% and 19.6%, respectively. According to the RCA test results, the SMS solution could not enhance the strength of the RCA; hence, the strength enhancement of MRAC was due to the improvement of the interface between the MRCA and the new mortar.

When the WG solution and SMS solution were used in combination to treat the RCA, the compressive strength and splitting tensile strength of the SMRAC increased by up to 34.8% and 32.8%, respectively. This means that the strength performance of the SMRAC2 was approaching or even surpassing that of the NAC. The enhancement of the strength was due to the combined reinforcement effect of the WG solution and SMS solution. The WG solution could improve the strength and reduce the water absorption of the RCA, while the SMS solution could enhance the interface between the RCA and the new mortar, and thus lead to a significant improvement in the RAC’s strength.

### 3.5. Capillary Water Absorption of Concrete

**Figure 12:**
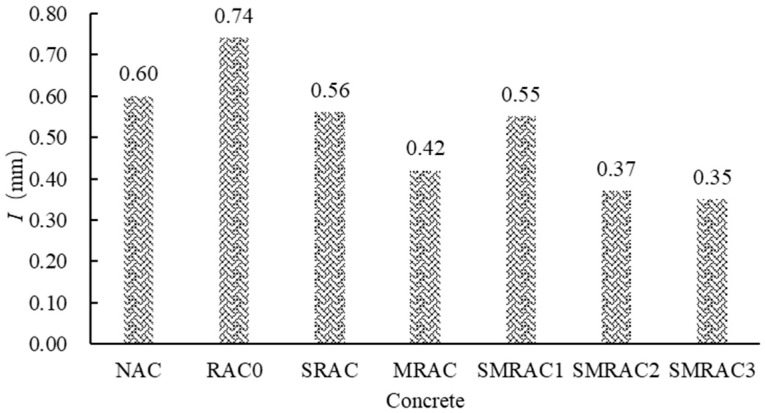
Results of capillary water absorption of concrete.

The results of the capillary water absorption (*I*) are shown in Figure 12. In concrete, during the water transmission process, the NCAs, with their low water absorption rate, block the pathways for moisture transmission, resulting in lower capillary water absorption of the NAC. Compared to the NAC, *I* of the RAC0 increased by 23.33%. This is because the water absorption of the untreated RCA (RCA0) is higher than that of the NCA. When the RCA0 was used in concrete to replace the NCA, more water could penetrate the RAC0, which negatively affected the durability of the RAC.

When the SRCA was used to replace the NCA, the *I* of the SRAC (*I*_SRAC_) showed a significant decrease. Compared to the RAC0, the *I*_SRAC_ decreased by 24.32%. This result is attributed to the reduction in water absorption of RCA caused by the WG solution treatment. When MRCA was used in concrete, the *I* of the MRAC (*I*_MRAC_) showed a further decrease, which was lower than that of the NAC. Compared to RAC0, the *I*_MRAC_ decreased by 43.24%. This is because the SMS solution can form a hydrophobic layer near the interface between the MRCA and the new mortar, effectively preventing the water transmission.

When the RCAs treated with WG and SMS were used in concrete, including SMRAC1, SMRAC2 and SMRAC3, the *I* of the SMRAC3 decreased by 52.70% compared to the RAC0 and by 41.67% compared to the NAC. This indicates that the combined treatment method can effectively combine the moisture-blocking effects of both the WG solution and the SMS solution. It is worth noting that *I*_SMRAC1_ was essentially equal to *I*_SRAC1_, indicating that the 8% SMS solution cannot form an effective hydrophobic layer near the interface between the MRCA and the new mortar. When the 10% SMS solution was used, *I*_SMRAC2_ showed a noticeable decrease. For the SMRAC3, compared to *I*_SMRAC2_, *I*_SMRAC3_ did not show a further significant decrease. This indicates that the 10% SMS solution was already effective in forming a good hydrophobic layer near the interface between the MRCA and the new mortar. Therefore, the 10% SMS solution is recommended.

### 3.6. Coulomb Electric Flux

**Figure 13:**
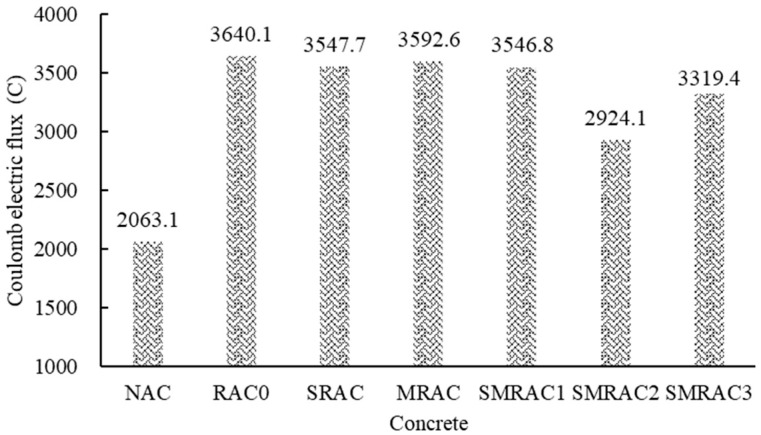
Results of coulomb electrical flux of concrete.

The results of the coulomb electrical flux (CEF) are shown in Figure 13. The CEF of the NAC was minimal, indicating that its ability to resist chloride ion penetration was the strongest. When the NA was replaced by the untreated RCA, the CEF of the RAC0 showed the highest value. This is because the adhered mortar of the RCA is porous, making it easier for water and chloride ions to penetrate the RAC0. When SRCA was used, the CEF of the SRAC was slightly affected. This is due to the modification effect of the WG solution, which could enhance the surface layer of the old mortar. However, it has little impact on the overall permeability of the concrete. According to the result for the MRAC, the influence of the SMS-solution-treated RCA on CEF was also insignificant. This is because the structure of the treated RCA was not changed and only a molecular film was formed. Under the saturation conditions, the hydrophobic molecular film could not effectively prevent the transmission of water. When the RCAs were treated with the WG solution combined with the SMS solution, the CEF of the SMRAC2 exhibited the most significant decrease, dropping by 19.67%. Nevertheless, it remained 41.73% higher than that of the NAC. This is due to the improvement of the pore structure within the SMRAC2, leading to the decrease in CEF. However, the porous old mortar of the treated RCA still cannot effectively prevent the penetration of chloride ions in a saturated state.

### 3.7. Nanoindentation

**Figure 14:**
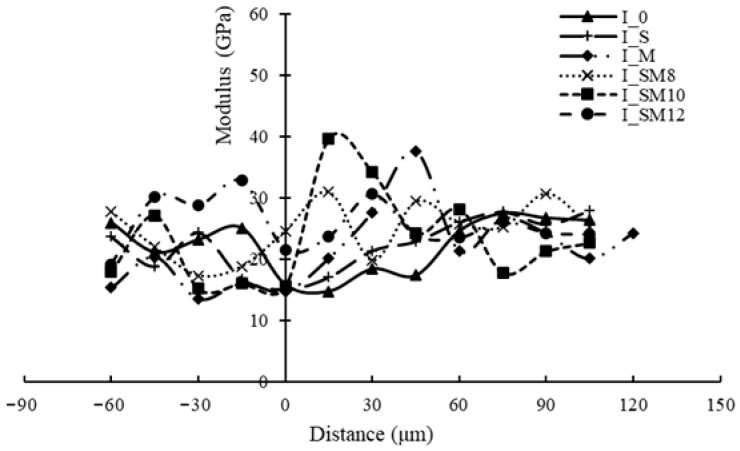
Nanoindentation results.

Figure 14 presents the nanoindentation results, revealing two distinct types of interface transition zones (ITZs). The first type exhibited a traditional weakening pattern, such as I_0, I_S. The modulus within the weakening ITZ was lower than that of the matrix mortar; similar results were also reported in [32]. The second type showed a strengthening pattern, such as I_M, I_SM8, I_SM10 and I_SM12. The modulus within the strengthened ITZ was higher than that of the matrix mortar. Mechanistically, when the SMS-modified RCA was incorporated into the new concrete system, the SMS coating not only formed a durable bond with the RCA substrate but also underwent chemical interactions with the fresh mortar. These reactions generated expansive products that effectively filled interfacial voids, resulting in the microstructural densification of the ITZ and consequent mechanical improvement. The I_SM10 specimen exhibited the maximum ITZ modulus, demonstrating that the optimal SMS solution concentration for performance enhancement is 10%. This phenomenon can be attributed to the concentration-dependent formation mechanism of hydration products. Increased SMS concentration (up to 10%) promotes the formation of expansive products within the ITZ, effectively reducing porosity through controlled volumetric expansion. Nevertheless, exceeding the critical concentration threshold (>10%) induces excessive products that generate internal stress concentrations, ultimately initiating microcracks that compromise the interfacial integrity and mechanical properties.

### 3.8. FTIR

**Figure 15:**
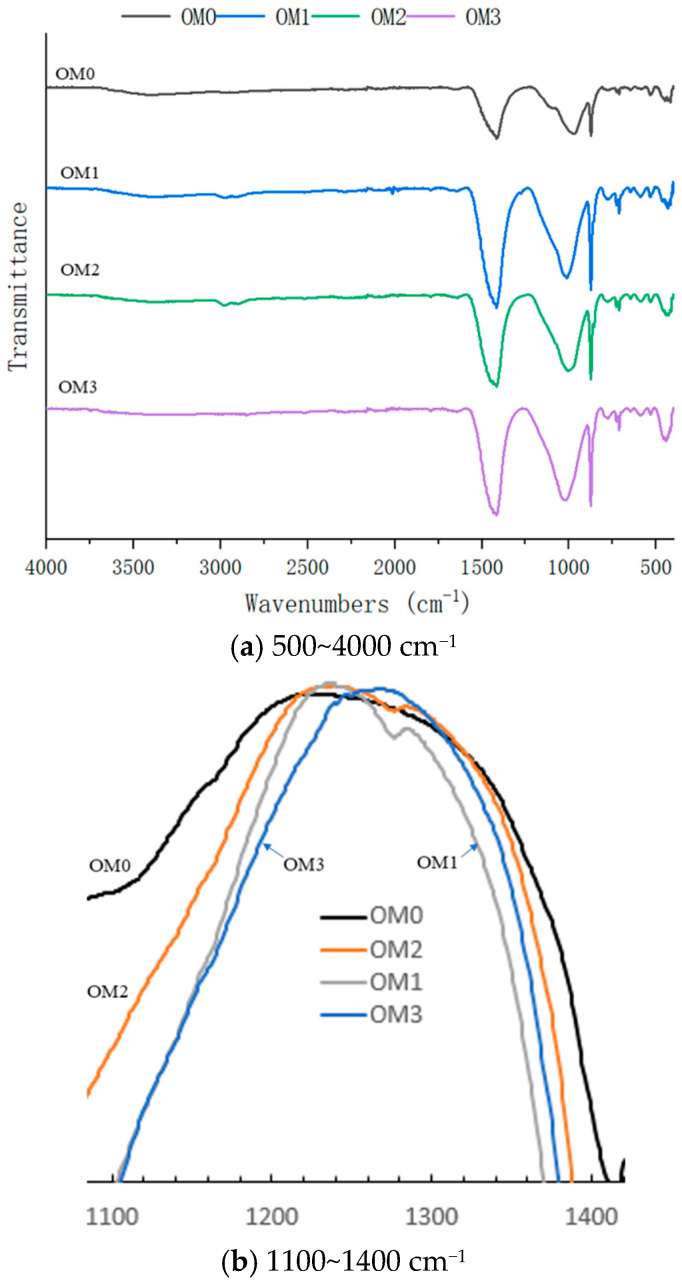
Infrared spectra of old mortars.

**Figure 16:**
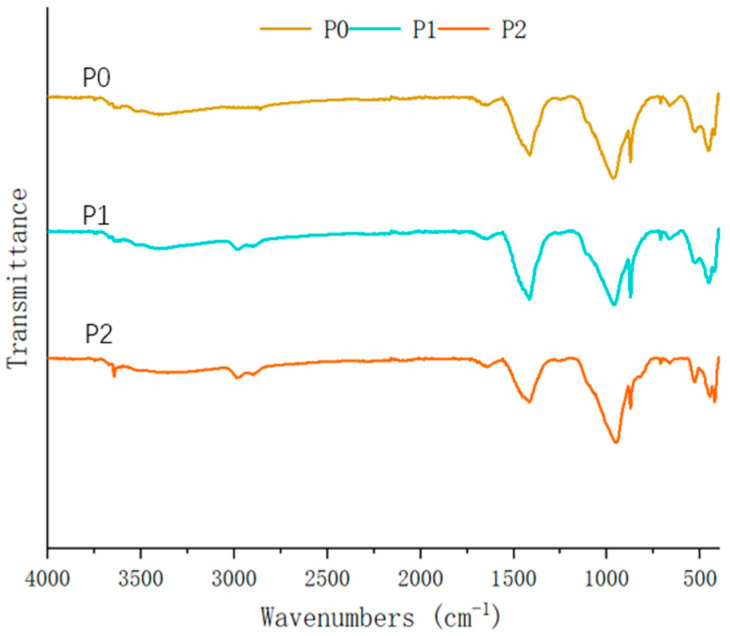
Infrared spectra of new cement pastes.

The FTIR results are shown in Figure 15 and Figure 16. As shown in Figure 15a, the Si-O absorption peak appears around 1016 cm^−1^, while the C-O absorption peaks are detected at approximately 1410 cm^−1^ and 870 cm^−1^, with no significant H-O absorption peak [50,51]. This suggests that Ca(OH)_2_ in the old mortar has reacted adequately with CO_2_ from the air [52,53]. The infrared spectra of different old mortars are essentially identical and similar to that of common cement concrete. This indicates that neither the WG solution nor the SMS solution significantly influenced the functional groups of the old mortar. Unlike other samples, the old mortar treated with the SMS solution showed an absorption peak around 2960 cm^−1^ and 1270 cm^−1^, as shown in Figure 15. This was the absorption peak of the methyl group [54], which was formed through the interaction between SMS and the old mortar. This chemical modification is responsible for imparting hydrophobicity to the treated old mortar.

The FTIR analysis of new cement pastes (Figure 16) revealed spectral characteristics that are fundamentally consistent with the reference sample (P0), indicating that the SMS solution’s interaction with the cement hydration products preserved the essential functional group chemistry of the hardened matrix. Notably, the characteristic absorption band near 2960 cm^−1^ appeared, which meant that the methyl group also existed in P1 and P2. In new cement paste systems, the predominant reaction involves the SMS solution interacting with CaO to form calcium methyl silicate, a chemical transformation that fails to impart hydrophobic properties to the cementitious matrix [45,55].

## 4. Conclusions

Based on the abovementioned results, the following conclusions can be drawn:

- The combined treatment using the WG solution and SMS solution could enhance the overall performance of RCAs, with the optimal effect achieved when the WG solution concentration was 40% and the SMS solution concentration was 10%. This combination significantly reduced the water absorption and crushing value and enhanced the apparent density of the RCA by improving the internal pore structure and the surface compactness, and by forming a hydrophobic film.
- The mechanical performance and water absorption of RAC prepared with treated RCA was markedly improved. The optimal combined treatment increased the compressive strength by approximately 35% and splitting tensile strength by over 20% compared to untreated RAC, while simultaneously reducing water absorption by nearly 46%, indicating a clear synergistic effect between WG and SMS.
- Contact angle, SEM, nanoindentation, and FTIR analyses confirmed that the combined treatment densified the interfacial transition zone (ITZ) and formed a stable hydrophobic layer. These mechanisms jointly contributed to the improvement in both mechanical strength and durability.
- The WG + SMS treatment provides a promising and eco-friendly approach for upgrading the performance of RCA and RAC, offering potential benefits for sustainable concrete production. However, further research is still needed to evaluate its long-term durability, cost-effectiveness, and industrial scalability before practical application.

## Figures and Tables

**Table 1:** Properties of raw RCA [35].

Property	Apparent Density (kg/m^3^)	Water Absorption (%)	Crushing Value (%)	Particle Size (mm)
Result	2687.3	5.81	15.27	9.5~19
Grade	I	III	II	-

**Table 2:** Main compositions of the cement [23].

Composition	CaO	SiO_2_	Al_2_O_3_	Fe_2_O_3_	MgO	K_2_O	Na_2_O
Percentage (wt.%)	59.3	20.5	6.3	4.1	2.0	0.3	0.2

**Table 3:** Experimental scheme for RCA.

Sample ID	Treatment Method	
	WG (%)	SMS (%)
RCA0	-	-
MRCA	-	10
SRCA	40	-
SMRCA1	40	8
SMRCA2	40	10
SMRCA3	40	12

**Table 4:** Concrete proportions (per m^3^) [36].

Coarse Aggregate/kg	Cement/kg	Sand/kg	Water/kg
1003	365	821	182.5

**Table 5:** Experimental scheme for concrete.

Sample ID	Coarse Aggregate Type
NAC	NA
RAC0	RCA0
SRAC	SRCA
MRAC	MRCA
SMRAC1	SMRCA1
SMRAC2	SMRCA2
SMRAC3	SMRCA3

**Table 6:** NI test scheme.

Sample ID	I_0	I_S	I_M	I_SM8	I_SM10	I_SM12
Aggregate type	RCA0	SRCA	MRCA	SMRCA1	SMRCA2	SMRCA3

**Table 7:** FTIR test scheme.

Sample ID	Description
OM0	Old mortar (untreated)
OM1	Old mortar (treated with SMS solution)
OM2	Old mortar (treated with WG solution + SMS solution)
OM3	Old mortar (treated with WG solution)
P0	New paste (no SMS was added)
P1	New paste (5% of water was replaced by the SMS solution)
P2	New mortar (75% of water was replaced by the SMS solution)

Remark: The concentrations of the SMS solution and the WG solution are 10% and 40%, respectively.

## Data Availability

The original contributions presented in this study are included in the article. Further inquiries can be directed to the corresponding author.
